# Mediterranean Diet and Cardiometabolic Diseases in Racial/Ethnic Minority Populations in the United States

**DOI:** 10.3390/nu10030352

**Published:** 2018-03-14

**Authors:** Mercedes Sotos-Prieto, Josiemer Mattei

**Affiliations:** 1 Department of Environmental Health, Harvard T.H. Chan School of Public Health, 677 Huntington Avenue, Boston, MA 02115, USA; 2 Department of Food Sciences and Nutrition, School of Applied Health Sciences and Wellness, Ohio University, Athens, OH 45701, USA; 3 Diabetes Institute, Ohio University, Athens, OH 45701, USA; 4 Department of Nutrition, Harvard T.H. Chan School of Public Health, 665 Huntington Avenue, Boston, MA 02115, USA

**Keywords:** Mediterranean diet, racial/ethnic minorities, diverse populations, cardiometabolic conditions, cardiovascular disease, type 2 diabetes, obesity

## Abstract

The Mediterranean diet (MedDiet) has been recommended to the general population by many scientific organizations as a healthy dietary pattern, based on strong evidence of association with improved cardiometabolic health, including lower risk of cardiovascular disease, diabetes, and obesity. However, most studies have been conducted in Mediterranean or European countries or among white populations in the United States (US), while few exist for non-Mediterranean countries or racial/ethnic minority populations in the US. Because most existing studies evaluating adherence to the MedDiet use population-specific definitions or scores, the reported associations may not necessarily apply to other racial/ethnic populations that may have different distributions of intake. Moreover, racial/ethnic groups may have diets that do not comprise the typical Mediterranean foods captured by these scores. Thus, there is a need to determine if similar positive effects from following a MedDiet are observed in diverse populations, as well as to identify culturally-relevant foods reflected within Mediterranean-like patterns, that can facilitate implementation and promotion of such among broader racial/ethnic groups. In this narrative review, we summarize and discuss the evidence from observational and intervention studies on the MedDiet and cardiometabolic diseases in racial/ethnic minority populations in the US, and offer recommendations to enhance research on MedDiet for such populations.

## 1. Introduction

The Mediterranean diet (MedDiet) has been recommended to the general population by many scientific organizations as a healthy dietary pattern [1,2] based on strong evidence that it improves cardiometabolic health [3,4,5]. The MedDiet was first described in the early 1960′s as a dietary pattern typical of Crete, most of Greece, and southern Italy [6]. Variations of the MedDiet exist in other countries bordering the Mediterranean Sea. However, common characteristics of the MedDiet include a high consumption of fruits, vegetables, olive oil as a main fat, whole grain, legumes, and nuts; moderate consumption of fish, poultry and dairy; and low intake of red meat, processed meats, and sweets; and wine in moderation [6]. 

Compelling evidence from meta-analyses showed an inverse association between consuming a MedDiet and all-cause mortality, cardiovascular disease (CVD) [5,7,8], and other cardiometabolic risk factors such as metabolic syndrome [9], type 2 diabetes (T2D) [10], and obesity [11]. In addition, clinical trials using the MedDiet have been successful in the primary [4] and secondary prevention of CVD [12]. However, most of the studies assessing the health benefits of the MedDiet have been conducted in Mediterranean or European countries [4,13,14,15,16] or among white populations in the United States (US) [17,18,19], while few exist for non-Mediterranean countries or racial/ethnic minority populations in the US [20]; that is, individuals identifying as Black or African American, American Indian or Alaska Native, Asian, Native Hawaiian or Other Pacific Islander, or Hispanic/Latino and who are underrepresented in research compared to the white racial majority [21,22], even though collectively all minority non-white groups account for over 40% of the U.S. population [23]. 

In epidemiological studies, the MedDiet has been defined using various criteria, with the most common definition being the revised MedDiet score by Trichopoulou et al. [16] or adaptions from this [24] that assigns positive scores for healthy intakes or no scores for unhealthy intakes of several typical Mediterranean food components as based on the population’s sex-specific median cutoffs. Because most existing studies evaluating adherence to the MedDiet use population-specific definitions, the associations between these diet scores as developed in white populations may not necessarily apply to other racial/ethnic populations that may have different distributions of intake. Moreover, racial/ethnic groups may have diets that do not comprise the typical Mediterranean foods reflected in these scores [20]. For example, olive oil, a traditional Mediterranean component, tends to be reflected in the scores as monunsaturated fatty acids:saturated fatty acids (MUFA:SFA) ratio, which may represent consumption of other types of oils. One study conducted in a multi-ethnic population in the US with 63% Hispanic and 20% Black participants reported lower consumption of MedDiet components (specifically, fruit, vegetables, legumes, fish, cereals, and MUFA) in comparison with other studies in European and American populations [25]. In the study, Hispanics, who were predominantly of Dominican heritage, showed greater adherence to MedDiet. The authors concluded that the dietary patterns of this group may not accurately reflect a true diet followed in the Mediterranean region. Similarly, a study conducted in US-mainland Puerto Ricans found that the MedDiet components represented in the Puerto Rican diet included foods not typically observed in the traditional Mediterranean patterns [20]. Finally, a study contrasting the Mediterranean Adequacy Index, which assesses how close a diet is to a healthful MedDiet, in 23 global populations, noted high index values mostly among men from southern Italy and the Greek islands, while considerably low values were noted among participants from Spanish-speaking Latin American countries [26].

Of note, racial/ethnic minority populations in the US present disparities in prevalence and incidence of cardiometabolic conditions, as we illustrate in the subsequent sections [27,28,29,30,31]. In search of understanding and curbing health disparities through healthier lifestyles, there is a need to determine if similar positive effects from following a MedDiet are observed in other non-Mediterranean or non-white populations, as well as to identify culturally-relevant foods and lifestyles reflected within Mediterranean-like patterns, that can facilitate implementation and promotion of such among broader racial/ethnic groups. In this narrative review, we summarize the evidence from observational (Table 1) and intervention studies on the MedDiet and major cardiometabolic diseases (i.e., diabetes, obesity, cardiovascular disease, and their biological risk factors) in racial/ethnic minority populations living in the US, describe the cultural adaptations of the original MedDiet from the intervention studies reported (Table 2), discuss the implications of following a MedDiet and offer recommendations to enhance research on MedDiet for such populations.

## 2. Evidence on Mediterranean Diet and Diabetes and Obesity 

### 2.1. Background

Evidence from large population-based studies and randomized clinical trials (RCTs) in predominately white (including European) populations have shown that adhering to a Mediterranean dietary pattern may prevent risk of T2D and obesity, as well as related metabolic outcomes [9,11,44,45,46,47,48,49]. A recent review of meta-analyses studies showed that adherence to MedDiet translated to a pooled relative risk of 0.83 (0.74, 0.93) in diabetes, and 0.82 (0.70, 0.96) in elevated waist circumference based on observational studies. Evidence from RCTs returned a pooled risk for diabetes of 0.70 (0.54, 0.91), and a mean difference in waist circumference of −0.51 (−0.65, −0.36) cm [50]. Postulated mechanisms include decreases in oxidative stress, inflammation, and insulin resistance mediated by Mediterranean food components [44]. These benefits in reducing obesity and diabetes would be valuable for some racial/ethnic populations in the US, for which prevalence and incidence of T2D and obesity remain disproportionately high. 

According to 2011–2012 National Health and Nutrition Examination Survey (NHANES) data, the age-standardized (95% CI) prevalence of diabetes was higher among non-Hispanic Blacks (21.8% (17.7%, 26.7%)), non-Hispanic Asians (20.6% (15.0%, 27.6%)), and Hispanics (22.6% (18.4–27.5%)), compared to non-Hispanic whites (11.3% (9.0–14.1%)); undiagnosed cases were higher among non-Hispanic Asians and Hispanics [51]. Reports using 2011–2014 NHANES data show similar disparities in obesity, for which non-Hispanics Black (48.1%) and Hispanic (42.5%) adults have higher prevalence, while non-Hispanic Asians (11.7%) have lower prevalence than non-Hispanic white adults (34.5%) [52]. Similar patterns for obesity prevalence have been reported for children and adolescents [52]. 

Variations in diabetes and obesity prevalence have been reported across specific ethnicities within a racial/ethnic group. For example, diabetes prevalence among Hispanic/Latino heritages ranged from 10.2% in South Americans to 18.0% in Dominicans and Puerto Ricans, and 18.3% in Mexicans [53]. Similar variations were noted for Asian ethnic groups living in the US, for which estimates ranged between 12.8% and 46.7% for overweight and 2.1–59.0% for obesity across specific ethnicities; diabetes was reportedly highest among those of Asian Indian heritage [54].

Despite the substantial evidence of the benefits of the MedDiet on metabolic diseases and of the disparities in such conditions by racial/ethnic minority groups, we noticed a scarcity of studies correlating MedDiet and diabetes- or obesity-related conditions in these populations. In the following section, we summarize the evidence from population-based, RCTs, and small trials assessing MedDiet on obesity and diabetes outcomes in racial/ethnic minority populations living in US. 

### 2.2. Population-Based Studies

The Multi-Ethnic Study of Atherosclerosis (MESA) was one of the first population-based prospective cohort studies reporting associations between MedDiet and diabetes-related outcomes in a multi-ethnic sample (white, African American, Hispanic, and Chinese) of men and women aged 45–84 years living in US [32]. The study defined a MedDiet score based on population-median cutoffs of ten food components (vegetables; whole grains; nuts; legumes; fruits; ratio of MUFA:SFA; red and processed meat; dairy products; fish; and alcohol) as assessed with a food frequency questionnaire (FFQ). Endpoints included fasting glucose and insulin at baseline and incident T2D at six-year follow-up. The study found that for the whole multi-ethnic cohort, a higher MedDiet score was associated with lower baseline mean insulin levels and lower glucose levels (the latter was attenuated after adjusting for waist circumference) but was not significantly associated with diabetes risk. However, the study did not stratify by race/ethnicity as the interaction terms with MedDiet were not significant. The study concluded that that observed associations were thus consistent across racial/ethnic groups.

A similar conclusion was reached by the Coronary Artery Risk Development in Young Adults (CARDIA) study that analyzed the association of a modified MedDiet score with 25-year incidence of metabolic syndrome among 18–30 years old African American and white adults [34]. The MedDiet was assessed with a diet history questionnaire at baseline, year 7 and year 20, and the score included whole grains, fruit, vegetables, fruit and vegetable juice, legumes, nuts, poultry, fish, eggs, coffee and tea, MUFA + polyunsaturated (PUFA):SFA, red and processed meat, dairy products, fried vegetables, refined grain, sauces, snack foods, sugar-sweetened beverages, diet beverages, and alcohol. The study did not stratify between the two racial groups, as no effect modification by race was detected. Their observations for the overall cohort was that incidence for metabolic syndrome, as well as its components of abdominal obesity, elevated triglycerides, and low HDL-C, was lower in those with higher MedDiet scores compared to lower scores. 

Of note, the Women’s Health Initiative, a US-wide longitudinal study of postmenopausal women aged 50–79 years, observed a significant association between highest quintile (healthiest) of the alternate Healthy Eating Index, which has some components similar to MedDiet but uses a different scoring system, compared to the lowest quintile, and diabetes risk only in whites (HR (95% CI) 0.74 (0.68–0.82)) and Hispanics (0.68 (0.46–0.99)), but not in Blacks (0.85 (0.69–1.05)) or Asians (0.88 (0.57–1.38)) [55].

A study that reported stratified ethnic-specific associations for MedDiet was the Multiethnic Study (MEC), a longitudinal study of diet and cancer among men and women aged 45–75 years living in Hawaii and California. The authors aimed to assess the association between four dietary indices using food and nutrients assessed from a FFQ, including a modified MedDiet as defined by Fung et al. [24] hat included vegetables without potatoes, fruits, whole grains, nuts, legumes, fish, red and processed meat, alcohol consumption, and MUFA:saturated fat, and T2D risk [35]. The study was conducted among whites, Native Hawaiians, and Japanese Americans, given their premise that cultural differences in dietary habits and biologic differences in metabolism may translate into differential associations across ethnic groups. The study found that a higher adherence to MedDiet was related to a 13–28% lower risk of T2D in white participants but not in other ethnic groups (HR (95% CI): 0.90 (0.84, 0.95) white men; 0.95 (0.88, 1.03) Native Hawaiian men; 0.98 (0.93, 1.02) Japanese American men; 0.93 (0.86, 1.00) white women; 0.97 (0.90, 1.05) Native Hawaiian women; 1.00 (0.95, 1.05) Japanese American women). The only significant associations for T2D noted for non-white ethnic groups were for the alternate Healthy Eating Index in Native Hawaiian women (0.93 (0.87, 0.99)) and the Dietary Approaches to Stop Hypertension (DASH) diet score in Japanese American women (0.93 (0.89, 0.97)), suggesting that food components captured by those scores may be more relevant for these ethnic groups [35]. The authors noted that the dietary scores were originally created and tested among European, white, or African American populations, which may not capture traditional foods consumed by Japanese Americans and Native Hawaiians.

A subsequent study contrasted five diet quality scores, including a MedDiet score adapted from Trichopoulou et al. [16] as assessed from a FFQ that captured culturally-appropriate foods and portions, with metabolic outcomes among Puerto Rican adults 45–75 years participating in the longitudinal Boston Puerto Rican Health Study [20]. The study detected significant associations between the MedDiet score and several outcomes measured at two-years, which were stronger than for the other diet quality scores: lower waist circumference (β coefficient ± SE: −0.52 ± 0.26); BMI (−0.23 ± 0.08); log-insulin (−0.06 ± 0.02); log-homeostasis model assessment of insulin resistance (−0.05 ± 0.02), and log-C-reactive protein (−0.13 ± 0.03). The authors concluded that the MedDiet score contains food components and weights relevant for cardiometabolic regulation for Puerto Ricans, as it likely captures the population’s dietary variation. Moreover, the authors assessed the traditional Puerto Rican foods consumed by individuals with high MedDiet as this would help promote familiar foods shown to be associated with favorable metabolic outcomes. The foods included vegetables (such as root crops and green bananas) and meats in homemade soups, orange juice, oatmeal, beans and legumes, fish (such as cod and canned tuna), whole milk, corn oil, and beer. Notably, while some components are present in the Mediterranean diet pattern, other foods are not, such as meats in homemade soups, orange juice, or whole milk, corn oil, or beer. The results support that essential components of the Mediterranean diet may improve cardiometabolic parameters but the pattern also includes population-specific traditional foods. 

Although not a US minority study, a nationwide, cross-sectional study among adults in Chile is one of the few assessing the benefits of the MedDiet on metabolic health in a Latin American population [56]. The study used the Chilean Mediterranean Diet Index (Chilean-MDI), which adapted and validated the MedDiet score criteria for use in Chile by incorporating local Chilean eating patterns and ingredients in a short, easy-to-implement, self-assessment online tool [56]. The authors reported an inverse association between adherence to the Chilean-MDI and prevalence of overweight, obesity, and metabolic syndrome, and proposed using adapted Mediterranean diets as a chronic disease risk management strategy in populations beyond the Mediterranean basin [56].

### 2.3. Randomized Clinical Trials and Interventions

Few interventions using the MedDiet for prevention or control of cardiometabolic parameters have targeted racial/ethnic minority participants. One example is the ¡Viva Bien! study, that randomly assigned 280 Latinas with T2D to usual care only or to usual care plus ¡Viva Bien! [57]. The program consisted of group meetings to promote the MedDiet, physical activity, stress management, supportive resources, and smoking cessation. The ¡Viva Bien! adaptation used a participant-focused iterative process that produced high satisfaction rate to adapt the dietary recommendations of the MedDiet guidelines to the Latina’s preferences, among other cultural tailoring [41,57]. Participants were instructed by a dietitian to consume healthy components of the MedDiet while limiting animal fat and controlling portion sizes to reduce energy intake. Latin American recipes were modified to conform to these guidelines while using traditional staple Latino ingredients and spices, and trying to cover the wide range of diverse Latin American foods. Educational, skill-based, and family support components were included for acceptance of the MedDiet adaptations, such as cooking demonstrations and MedDiet potluck dinners. The program showed better, albeit modest, decreases in hemoglobin A1c compared to participants in usual care at six-months, with lessened but significant decreases at 12-months, based on intention-to-treat analysis [57].

We could not find other MedDiet trials for metabolic improvement deliberately crafted for minority groups. The Heart Healthy Lenoir Project, a 3-phase weight loss and maintenance intervention promoting a Mediterranean-style dietary pattern and increased walking, had 65% of their participants identified as African American [42,58]. However, this ethnic composition was likely due to the location of the study in Lenoir County, North Carolina, which has a high African American population as well as documented disparities in heart disease and stroke. The study recruited participants 18 years and older with an interest in improving diet and physical activity behaviors to reduce CVD risk. The dietary intervention was based on the PREDIMED trial [4] with an emphasis on improving the quality of fat and carbohydrate, and the adaptations responded to regional cuisine than to racial preferences, thus named the Med-South Diet [42]. The authors aimed to craft the intervention for both minority and non-minority participants with the goal of decreasing health disparities. The study reported improvements in Med-South Diet score, generally maintained through 24-month follow-up; those in phase 2 and 3 weight loss interventions had significant but modest weight reductions at 12-months and 24-months [42]. Of note, the authors reported mostly similar outcomes for African American and white participants by treatment, although weight loss at 24-months was significant for all African Americans but not for white participants (mean (95% CI) change in kg: −1.8 (−2.8, −0.8) vs. −1.0 (−2.9, 0.8). This may suggest that while the MedDiet may equally generate lifestyle and physiologic changes for African American and white participants, African Americans may have a marginally better long-term weight loss response.

Subsequently, Embree et al. assessed weight changes among Heart Healthy Lenoir Project participants by diabetes status [58]. They showed larger improvements in the summary score for drinks, desserts, and snacks consumption among African-American participants with and without diabetes compared with whites. White participants with diabetes had greater weight loss than African-American participants with diabetes, with no differences by race for those without diabetes. Changes in hemoglobin A1c were not significant, although African Americans with diabetes had a slightly larger decrease than white participants with diabetes. 

## 3. Evidence on Mediterranean Diet and Cardiovascular Disease

### 3.1. Background

Evidence from previous and updated meta-analyses showed consistent association between the adherence to the MedDiet and mortality, and CVD [7,8,50,59,60,61,62]. Data from 16 observational studies showed that a two-point increase in adherence to the MedDiet was associated with a 13% (95% CI: 10%, 15%) lower risk of CVD events [7,60]. A more recent meta-analysis showed that compared to the lowest adherence to MedDiet, the greatest adherence was associated with 24% (17%, 32%) lower incidence of CVD, 28% (14%, 40%) lower coronary heart disease mortality, 37% (17%, 46%) lower myocardial infarction mortality, and 24% (4%, 40%) lower stroke mortality [8]. The pooled analysis by individual components of the MedDiet attributed the strongest effects to olive oil, fruits, vegetables, and legumes consumption [8]. Despite the decline in CVD mortality, CVD remains the leading cause of death in the United States [28]. As previously mentioned, some racial and ethnic minorities are disproportionally affected [27,28,29,30,31]; with 33% higher CVD age-adjusted death rates for Blacks than for the overall population in U.S [28]. Life expectancy of African American is around 3 years shorter than whites [27]. In addition, according to the Centers for Disease Control and Prevention, 36% of American Indians/Alaska Natives under 65 years die from heart disease compared to 17% of the overall U.S. population [63]. Estimates from the American Heart Association state that between 2007 and 2013, CVD and stroke death rates decreased 12.5% and 16.1% respectively in non-Hispanic whites; 16.5% and 20.2% in non-Hispanic Blacks; 18.1% and 17.3% in Hispanics; 15.0% and 19.6% in non-Hispanic Asian and Pacific Islanders; and 11.3% and 22.5% in non-Hispanic American Indian or Alaska Natives [28]. While adherence to MedDiet could help improve the CVD statistics, it is important to understand the effects of MedDiet across different ethnicities. In the following section we summarize the evidence from observational studies and RCTs assessing the MedDiet and CVD in racial/ethnic minority populations living in US [28].

### 3.2. Population-Based Studies

Observational studies analyzing the association between the MedDiet and CVD among minorities in US are scarce, with inconsistent results on the magnitude of effect by race/ethnicity. The MEC study included white, African American, Native Hawaiian, Japanese American and Latino adults. Harmon et al. evaluated the adherence to different dietary patterns including the MedDiet and total and cause-specific mortality [36]. After 13–18 years of follow-up, the MedDiet showed an overall association with CVD mortality, yet the strength of the association varied by ethnicity. Being on the fifth quintile (highest adherence group) of the MedDiet, compared to the first quintile (lowest adherence group) was associated with lower risk of CVD mortality only in African American men (HR (95% CI): 0.75 (0.62, 0.90)), and women (0.82 (0.70, 0.97)) and Japanese American men (0.80 (0.68, 0.94)) and women (0.72 (0.59, 0.87)) but not in Latino or Native Hawaiian men and women. Results were slightly more robust among whites participants (0.70 (0.59, 0.84) in men, 0.77 (0.62, 0.95) in women), for which the MedDiet score used in the study was developed. The mis- or under-representation of unique cultural dietary aspects of Native Hawaiians and Latinos may partly explain the lack of associations among these ethnic groups. Reduced sample size may also account for the null results.

The REasons for Geographic and Racial Differences in Stroke (REGARDS) study, a longitudinal cohort of Black and white men and women ≥45 years of age (*n* = 21,423) investigated the association of the MedDiet with all-cause and cause-specific mortality (6.25 years follow-up) [37]. The adherence to the MedDiet was evaluated by 11 components (vegetables, fruits, lean meats, fish, nuts, MUFA:saturated fats, red and processed meats, sodium, dairy foods, grains and starches, and alcohol) and based on a participant’s quintile of intake [37]. The researchers found that compared with those in the lowest quintile of MedDiet score (lowest adherence), participants with the highest MedDiet adherence (highest adherence) had 32% lower risk of CVD mortality (95% CI: 47%, 12%) and the association across race were similar. The authors noted that they used relative instead of absolute cutoff scoring, which may lead to fewer participants being truly concordant with a MedDiet and may underestimate the effects [37].

The Northern Manhattan Study (NOMAS) study is a prospective cohort study in New York city that included 63% Hispanics, 20% non-Hispanic Blacks, and 15% non-Hispanic whites with the aim to identify stroke risk factors (*n* = 2568; median follow-up 9 years) [25]. The study evaluated adherence to the MedDiet following the original Trichopoulou et al. [16] Compared with those in the first quintile, participants in the top quintile of the MedDiet had 28% (95% CI: 4%, 46%) lower risk of combined vascular events (ischemic stroke, myocardial infarction, and vascular death). Individually, the MedDiet was inversely associated with vascular death only. Interestingly, the food components driving the observed associations were alcohol, fish, and legumes—foods that may be more represented in the diets of Hispanics and Blacks. Nonetheless, there was no significant interaction between race-ethnicity and MedDiet score in relation to the vascular events. The authors concluded that while a MedDiet may protect against vascular outcomes in this multi-ethnic cohort, the dose of the MedDiet in this predominantly minority population may not be sufficiently high or broadly distributed to detect a strong protective association with vascular events, even for those with the healthiest intake [25]. Also in NOMAS, a cross-sectional analysis showed that greater adherence to MedDiet was associated with lower left ventricular mass (1.98 g lower per 1-point of the diet score), a risk factor for CVD [38]. Again, no significant interactions were found by race/ethnicity. Similarly, MESA showed that a higher adherence to the MedDiet was cross-sectionally associated with modestly better left ventricular structure and function; however, the study did not stratify by race/ethnicity nor reported testing for interaction terms [33]. NOMAS also reported that the MedDiet was not associated with carotid intima media thicknesses, a marker of carotid atherosclerosis, in the whole multi-ethnic cohort [39]. In the study, the only MedDiet component that was independently associated with plaque thickness or area was vegetable consumption, showing an inverse association with plaque thickness. The authors did stratify by race, finding no association between MedDiet and plaque thickness nor area in African Americans or whites. For Hispanics, an inverse association was found between MedDiet adherence and the 75th percentile of plaque thickness (beta-coefficient, (95% CI): −0.0906 (−0.1541, −0.0271) change in mm) [39]. Reasons underlying the potential effect modification by race/ethnicity are not known, but could relate to subtle differences in the types of foods consumed across race/ethnic groups or related health behaviors. The authors noted that the study may have been underpowered to detect differences for African Americans and whites [39].

Finally, the Washington Heights-Inwood Community Aging Project (WHICAP) study included 4308 participants aged 65 or older living in Manhattan from three ethnicities (white, Hispanics, and African Americans). In a cross-sectional analysis, the authors evaluated the association of MedDiet as defined by Trichopoulou et al. and leukocyte telomere length (LTL), a marker of higher all-cause mortality and age-related conditions such as CVD, diabetes, and dementia [40]. They found that the MedDiet was associated with LTL only in whites but not among Hispanics or African Americans. Although no other study analyzed the relation between MedDiet pattern and telomere length as a marker of CVD across minority populations, in MESA, older age had a larger effect with shorter telomere length in African American and Hispanics regardless of diet and other lifestyles; more research is needed on this field [64]. Generally, authors of the aforementioned articles noted that because the scoring criteria rely on the distribution of intake rather than the absolute intake, few participants had a pattern resembling the traditional MedDiet. 

### 3.3. Randomized Control Trials and Interventions

Randomized control trials assessing the MedDiet in minority populations are also scarce. The ¡Viva Bien! study that randomized 280 Latinas with T2D to receive usual care or a culturally-adapted intervention based on the promotion of MedDiet al.ong with other lifestyle recommendations was specifically targeted to this underserved population at high risk for heart disease [41,57]. After a six months follow-up, participants in ¡Viva Bien! improved the calorie intake from saturated fat and the 10-year coronary heart disease risk score compared to those in usual care (mean score (SD): 9.1 (6.5) vs. 11.5 (7.9) risk score, in intention-to-treat analysis). Generally, the effects at 12 months decreased [57]. This study showed the importance of culturally-tailored interventions to modify health behaviors and CVD-risk among minority T2D patients.

The Heart Healthy Lenoir Project trial has shown favorable CVD-related outcomes from a MedDiet and lifestyle intervention as described above, despite not being targeted for a minority population or for CVD prevention [42]. The study showed greater overall reductions in systolic and diastolic blood pressure for all African American vs. white participants at 24-months (mean change in mmHg (95% CI): −8.4 (−11.8, −5.1) vs. −4.1 (−8.5, 0.4) for systolic; −7.2 (−9.2, −5.1) vs. −5.4 (−7.6, −3.3) for diastolic); other lifestyle and physiologic outcomes tended to be similar by race [42]. Results by treatment arm tended to be similar for both races. The authors mentioned that an important aspect to help keep adherence to the adapted MedDiet was that foods typical in the southern diet were allowed despite their high total fat content. The adaptation aligns with recent evidence against a role of dietary fats in CVD risk and mortality, focusing instead on substituting healthy dietary fat such as those recommended by MedDiet and DASH [65,66]. The results may be relevant for potential cultural adaptations of MedDiet for CVD-prevention interventions in African American adults living in the southern US.

*EnForma* pilot study (*n* = 36) is an adaptation of the previously tested and evidence-informed lifestyle intervention based on Mediterranean-style dietary pattern (Mediterranean diet version of the Heart Healthy Lenoir Project [42]) aiming to reduce CVD risk among low-income Hispanics Americans (mostly Mexican) using bilingual and bicultural research [43]. The major focus of the intervention was to improve consumption of high quality fats (MUFA and PUFA, from fish and other plant foods) and high quality carbohydrates (fruits, vegetables and whole grains). The authors followed and adapted 9 out of 13 recommendations from the PREDIMED study (group in the intervention arm) (Table 1). Specific adaptations from the Heart Healthy Lenoir intervention were included in this study after conducting a focus group that suggested revisions for culturally-relevant pictures, food terms, and culinary preferences. They also promoted engagement and retention throughout the intervention. During the first visit, participants received tailored counseling on oils, dressings, nuts, fish, and meats. During the second visit, participants were counseled on drinks, desserts, fruits and vegetables, grains, and beans. After a follow-up of 3 months, the *EnForma* had high engagement (92% completed the intervention and follow-up measures), acceptability (100%), and perception of usefulness (85–100%). The mean dietary fat quality score improved by 0.5 units (95% CI: 0.0, 1.1) and the mean fruit and vegetable servings/day improved by 0.7/day (95% CI: 0.1, 1.3). While the study did not assess biological outcomes, demonstrating participation and engagement from Latinas in a MedDiet intervention can boost its promotion for use in RCTs for CVD-prevention among Latino communities and stimulate researchers to test feasibility among other racial/ethnic minorities. 

## 4. Conclusions and Recommendations

It is valuable to understand the role of the MedDiet in preventing and controlling cardiometabolic diseases among racial/ethnic minorities living in the US, in light of the strong evidence in support of consuming foods within the Mediterranean pattern towards disease prevention and of the evidence of multiple CVD and T2D-related disparities presented by minority populations. Nonetheless, we found a small number of research studies on this topic exclusively conducted, or reporting on, minority groups. The few existing studies yielded inconsistent results on the association between MedDiet and cardiometabolic outcomes across racial/ethnic groups. In general, studies reported no effect modification by race/ethnicity and concluded that the observed benefits of MedDiet on T2D, obesity, and CVD outcomes pertained similarly to all groups. Among studies that stratified by race/ethnicity a priori, higher adherence to MedDiet showed significant results on lower T2D risk for whites only, better cardiometabolic profile among Puerto Ricans, lower plaque thickness among Hispanics, and lower CVD mortality for African Americans and Japanese Americans; other associations were null. MedDiet interventions seemed to improve glycemic control and CVD risk among Latinas with diabetes as well as weight and blood pressure declines, especially among Blacks.

A challenge of some of the population-based studies examined in this review is the small sample size of the minority groups being studied. Underpowered statistical tests likely contributed to the null associations for some specific race/ethnic groups, or for effect modification testing. Substantiated a priori stratification by race/ethnicity due to differences in food choices and distributions or higher p-values for interaction (i.e., *p* < 0.1) may be considered. Other studies with large sample size have suggested that the strength of diet-disease associations does vary by race/ethnic group. For example, the large prospective Nurses’ Health Study I and II reported that a higher score of a dietary diabetes risk reduction score was inversely associated with risk of T2D in all racial and ethnic groups evaluated (whites, Blacks, Hispanics, and Asians) with slight variations, and the absolute risk difference was greater in minority women [67]. Variations in the strength of the association between diet quality scores and cardiometabolic outcomes have been reported even across individual Hispanic/Latino heritages [68]. This suggests that some food components may matter more for one group than for others; thus testing the MedDiet in well-powered studies may determine if it does indeed have varying degrees of cardiometabolic benefits across race/ethnic groups.

Another limitation noted in the literature was that few studies analyzed data on Asian or Native American heritages; most were Blacks and Hispanics and some multiethnic cohorts. Few studies further analyzed specific ethnic groups within boarder racial/ethnic categories despite indications that dietary cultural preference and other lifestyle factors related to the MedDiet may translate into differential associations or may require individual tailoring in interventions. Existing large cohort studies may provide an opportunity in testing race/ethnicity variations in MedDiet studies. Minority-exclusive cohort studies such as Jackson Heart Study (African Americans), Strong Heart Study (Native Americans), and the Hispanic Community Health Study/Study on Latinos also provide an opportunity to study the MedDiet, although accruing incident diabetes and CVD cases may be a limitation. Whenever possible, heritage-specific analysis should be conducted.

The MedDiet is rooted on the culinary and lifestyle preferences of the Mediterranean-bordering countries and many authors of the articles reviewed here noted that the dietary scores were originally created and tested among European or white populations, which may not capture traditional foods consumed by diverse groups. However, other studies have adapted scores and/or interventions effectively to capture Mediterranean-like factors of the diverse population under study; thus, even for populations with different staple foods, a MedDiet-like may be identified. Such was the case in the Boston Puerto Rican Study that characterized traditional Puerto Rican foods most often consumed within each component of the MedDiet [20] and the Chilean study that adapted a MedDiet score specifically to the culinary preferences of the country [56]. Conversely, the variation in scores and definitions due to some adaptions precludes from directly contrasting the level of adherence to MedDiet across populations. We, and others, have previously noted that the scoring and grouping of food components used to define a diet quality score may influence associations with health outcomes in minority cohorts [20,69,70]. Yet, using population-based cutoffs as commonly done with MedDiet scores may better capture the distribution of intake of food components that may not be as represented in diverse populations. Thus, the MedDiet score may be used with proper modifications in such groups. It is recommended to identify the cultural foods that are represented within the MedDiet food groups, as this can have translational applications. It would be also useful to contrast the results of adapted MedDiet scores against the original definition for comparability. 

The MedDiet has been perceived as more expensive [71] and it has been shown that the CVD-protective effects are sustained only for individuals at higher socioeconomic levels [72]. Thus, adhering to the MedDiet may be less affordable to racial/ethnic groups for which socioeconomic disparities are well documented. However, choosing Mediterranean-like foods may be feasible within a budget-conscious and culturally-appropriate diet. For example, the Boston Puerto Rican Study identified some inexpensive food alternatives: beans, cod and canned tuna, beer, and corn oil [20]. An analysis of the energy and nutrient density in relation to both energy and nutrient cost of typical MedDiet foods concluded that some of the components were affordable and socially acceptable (i.e., grains, pulses, legumes, nuts, vegetables and both fresh and dried fruit) [73]. These concerns should be kept in mind when designing MedDiet interventions for minority populations. The few trials analyzed here appropriately adapted the original PREDIMED diet to include culturally-appropriate, familiar, affordable foods and dietary behaviors to the target population. While adaptations may deviate from the original MedDiet definition, achieving acceptability, adherence, and eventual biological improvements is of priority. Along with intervening with communities, we should educate public health officials and the clinical workforce, especially those caring for minority populations, on the cardiometabolic benefits of the MedDiet. Finally, the MedDiet is part of a holistic way of life, and further studies may consider the health effect of other non-dietary behavioral factors such as traditional Mediterranean physical activity and sleeping patterns. 

We should note some limitations of our review. First, although we did a prudent search of the literature, we did not conduct a systematic review. Most studies reported here were conducted among adult populations; less is known about the role of the MedDiet in children and adolescents. We only discussed studies that reported results for individual races/ethnicities or a test for interactions; there might be other multiethnic studies studying MedDiet but not reporting stratified results. We limited the review to association of MedDiet and cardiometabolic outcomes; however, MedDiet may have benefits on other health-related outcomes in minority populations. For example, in the MEC study, the MedDiet was associated with lower colorectal mortality only in African-American women, not other races/ethnicities [74]. The Health, Aging and Body Composition prospective cohort study, comprised of 38.2% Black participants ages 70–79 years, reported that stronger adherence to the MedDiet was associated with lower rate of cognitive decline at 8 years follow-up among Blacks but not whites [75]; while the Boston Puerto Rican Health Study showed an association with higher cognitive score and lower cognitive impairment [76]. Tangney et al. reported no effect modification by white-Black race in the significant inverse association between MedDiet and cognitive decline in the Chicago Health and Aging Project [77]; similarly, there was no race interaction for the association between MedDiet and reduced incident cognitive impairment in REGARDS; [78] suggesting equal benefits for both racial groups. In NOMAS, the MedDiet was associated with better incident kidney function [79].

In conclusion, the small number of studies on MedDiet and cardiometabolic disease in racial/ethnic minority populations in the US prompts us to list recommendations for enhancing research and practice on this field (Box 1). Population-based studies need to continue building an evidence-base on the role of the MedDiet on cardiometabolic health among diverse populations. Public health promotion of MedDiet as a whole and its food components can also be directed at minority communities, especially as the studies reviewed here showed that, in general, MedDiet scores tended to be lower in minorities than in white/European populations. Notably, the studies showing significant beneficial associations in minority populations suggest that even at low adherence to this pattern, a healthy MedDiet may protect against cardiometabolic diseases. Adhering to MedDiet for gaining cardiometabolic benefits seems to be feasible and acceptable among minority populations given the proper cultural adaptations. MedDiet has been recommended to the general population by the Dietary Guidelines for Americans [2] and the American Heart Association [65], and promoting culturally appropriate MedDiet among diverse minority populations in the US may lessen the cardiometabolic disparities in these communities.

Box 1 **Recommendations to improve research and practice on the role of the Mediterranean diet (MedDiet) on cardiometabolic disease among racial/ethnic minority populations in the USA.**

*Population-based studies:*

- Assess MedDiet and cardiometabolic outcomes in existing multiethnic or minority-exclusive cohorts
- Augment multiethnic or minority-exclusive studies that can assess diet and cardiometabolic health
- Report interactions and/or stratified results in multi-ethnic studies
- Report results on subgroups or specific heritages when possible
- Adapt scores (food components) if necessary, although this may limit comparability
- Use population-based cutoffs to reflect the distribution and intake of the minority population
- Identify the list of traditional foods that comprise the MedDiet within the minority population
- Consider other Mediterranean lifestyles (physical activity patterns, sleeping habits)

*Interventions and practice:*

- Culturally-adapt MedDiet using traditional staple foods and ingredients as well as cooking and dietary behaviors of the culture
- Deep-tailor the intervention using other educational, skill-building, and social support strategies
- Assess feasibility and engagement of adhering to MedDiet before a clinical trial
- Educate and train public health and clinical staff (including those who work with minorities) on the benefits and applications of MedDiet
- Promote MedDiet at the public health level, targeting minority communities with culturally-appropriate messages and approaches

## Figures and Tables

**Table 1:** Summary of population-based studies assessing association of Mediterranean diet and cardiometabolic outcomes in minority racial/ethnic populations in the USA.

Study and Reference	Mediterranean Diet Construct	Race/Ethnicities	Sample Size and Age	Key Findings
Multi-Ethnic Study of Atherosclerosis (MESA) [32]	Sum of population sex-specific median of 9 groups: vegetables; whole grains; nuts; legumes; fruits; MUFA:SFA; red and processed meat; dairy; fish; alcohol	Whites, African Americans, Hispanics, and Chinese	45–84 years *n* = 5390	Higher MedDiet score was associated with lower baseline mean insulin levels and lower glucose levels but was not significantly associated with diabetes risk. No significant interaction by race.
Multi-Ethnic Study of Atherosclerosis (MESA) [33]	Sum of population sex-specific median of 9 groups: vegetables; whole grains; nuts; legumes; fruits; MUFA:SFA; red and processed meat; dairy; fish; alcohol	Whites, African Americans, Hispanics, and Chinese	45–84 years *n* = 4497	MedDiet associated with modestly better left ventricular (LV) structure and function (For each +1-U difference in score: LV volume was 0.4 (95% CI: 0.0, 0.8 mL) higher, the stroke volume was 0.5 (95% CI: 0.2, 0.8 mL) higher, and the ejection fraction was 0.2 percentage points (95% CI: 0.1, 0.3) higher. The study did not stratify by race/ethnicity nor reported testing for interaction.
Coronary Artery Risk Development in Young Adults (CARDIA) [34]	Sum of population median of: whole grains, fruit, vegetables, fruit and vegetable juice, legumes, nuts, poultry, fish, eggs, coffee and tea, MUFA+PUFA:SFA, red and processed meat, dairy products, fried vegetables, refined grain, sauces, snack foods, sugar-sweetened beverages, diet beverages, alcohol	African Americans and whites	18–30 years *n* = 4713	For the overall cohort, hazard ratio for metabolic syndrome 0.67 (0.49, 0.90) vs. 0.82 (0.67, 1.01), as well as incidence of its components of abdominal obesity (41.9 vs. 59.4%), elevated triglycerides (21.6 vs. 37.3%), and low HDL-C (59.3 vs. 68.4%), was better in those with higher MedDiet scores (top quintile) compared to lower scores (lowest quintile). No significant interaction by race.
Multiethnic Study (MEC) [35]	Sum of population median of 10 groups: vegetables without potatoes, fruits, whole grains, nuts, legumes, fish, red and processed meat, alcohol consumption, MUFA:SFA	Whites, Native Hawaiians, and Japanese Americans living in Hawaii and California	45–75 years *n* = 89,185	Higher adherence to MedDiet was related to a 13–28% lower risk of T2D in white participants but not in other ethnic groups (HR (95% CI): 0.90 (0.84, 0.95) white men; 0.95 (0.88, 1.03) Native Hawaiian men; 0.98 (0.93, 1.02) Japanese American men; 0.93 (0.86, 1.00) white women; 0.97 (0.90, 1.05) Native Hawaiian women; 1.00 (0.95, 1.05) Japanese American women).
Multiethnic Study (MEC) [36]	Sum of population median of 10 groups: vegetables without potatoes, fruits, whole grains, nuts, legumes, fish, red and processed meat, alcohol consumption, MUFA:SFA	White, African Americans, Native Hawaiians, Japanese Americans and Latinos	45–75 years *n* = 215,782	The MedDiet was associated with lower risk of CVD mortality only in whites participants (0.70 (0.59, 0.84) in men, 0.77 (0.62, 0.95) in women); African American men [HR (95% CI) 0.75 (0.62, 0.90)], and women [0.82 (0.70, 0.97)] and Japanese American men [0.80 (0.68, 0.94)] and women [0.72 (0.59, 0.87)] but not for in Latino or Native Hawaiian men and women.
Boston Puerto Rican Health Study [20]	Sum of sex-specific energy-adjusted population median for 9 components: vegetables, fruits, whole grains, nuts and legumes, meat, fish, dairy products, MUFA: SFA, and alcohol	Puerto Ricans living in Boston	45–75 years *n* = 1194	A higher MedDiet score was associated with 2-years lower waist circumference (β coefficient ± SE: −0.52 ± 0.26); BMI (−0.23 ± 0.08); log-insulin (−0.06 ± 0.02); log-homeostasis model assessment of insulin resistance (−0.05 ± 0.02), and log-C-reactive protein (−0.13 ± 0.03). Traditional foods consumed at high MedDiet included vegetables (e.g., root crops, green bananas) and meats in homemade soups, orange juice, oatmeal, beans, legumes, fish (e.g., cod, canned tuna), whole milk, corn oil, beer.
Racial Differences in Stroke (REGARDS) [37]	Sum of population-based quintiles of 11 components: vegetables, fruits, lean meats, fish, nuts, MUFA:SFA, red and processed meats, sodium, dairy foods, grains and starches, and alcohol	Black and white men and women	≥4 years *n* = 21,423	Compared with those in the lowest MedDiet score quintile, participants with the highest MedDiet adherence had 32% (95% CI: 47%, 12%) lower risk of CVD mortality after 6.25 years of follow-up. The associations were similar across race.
Northern Manhattan Study (NOMAS) [25]	Sum of sex-specific energy-adjusted population median for 9 components: fruits and nuts, vegetables, legumes, cereals and grains, fish, meat, dairy products, MUFA:SFA alcohol	Hispanics, non-Hispanic Blacks, and non-Hispanic whites from New York city	>40 years *n* = 2568	Compared with those in the first MedDiet score quintile, participants in the top quintile had 28% (95% CI: 4%, 46%) lower risk of the combined vascular events (ischemic stroke, myocardial infarction, and vascular death). Diet only was inversely associated with vascular death only. No significant interaction by race-ethnicity.
Northern Manhattan Study (NOMAS) [38]	Sum of sex-specific energy-adjusted population median for 9 components: fruits and nuts, vegetables, legumes, cereals and grains, fish, meat, dairy products, MUFA:SFA, alcohol	Hispanics, non-Hispanic Blacks, and non-Hispanic whites from New York city	>40 years *n* = 1937	Greater adherence to MedDiet was associated with lower left ventricular mass (1.98 g lower per 1-point of the diet score). Non-significant interactions by race/ethnicity.
Northern Manhattan Study (NOMAS) [39]	Sum of sex-specific energy-adjusted population median for 9 components: fruits and nuts, vegetables, legumes, cereals and grains, fish, meat, dairy products, MUFA:SFA, alcohol	Hispanics, non-Hispanic Blacks, and non-Hispanic whites from New York city	>40 years *n* = 1374	MedDiet was not associated with carotid intima media thicknesses in the whole multi-ethnic cohort. No association between MedDiet and plaque thickness nor area in African Americans or whites. For Hispanics, an inverse association was found between MedDiet adherence and the 75th percentile of plaque thickness (beta-coefficient, (95% CI): −0.0906 (−0.1541, −0.0271) change in mm).
Washington Heights-Inwood Community Aging Project (WHICAP) [40]	Sum of sex-specific energy-adjusted population median for 9 components: fruits and nuts, vegetables, legumes, cereals, fish, meat, dairy products, MUFA:SFA, alcohol	White, Hispanics, and African Americans living in Manhattan	≥ 65 years *n* = 4308	MedDiet score was associated with leukocyte telomere length only in whites (β = 48.3) but not among Hispanics or African Americans.

Note: All studies used a validated self-administered food frequency questionnaire (FFQ) for dietary assessment except CARDIA (Coronary Artery Risk Development in Young Adults) that used a diet history questionnaire. The following FFQ were used: MESA (Multi-Ethnic Study of Atherosclerosis): 127-item FFQ; MEC (Multiethnic Study): a quantitative FFQ; BPRHS (Boston Puerto Rican Health Study): a semi-quantitative FFQ; REGARDS: a Block FFQ; NOMAS (Northern Manhattan Study): a modified Block National Cancer Institute FFQ; WHICAP (Washington Heights-Inwood Community Aging Project): Willett’s semi-quantitative FFQ. Abbreviations: MedDiet: Mediterranean Diet; MUFA: Monounsaturated fats; PUFA: Polyunsaturated fats; SFA: Saturated fats; LV: Left ventricular; HR: Hazard Ratio; CVD: Cardiovascular disease.

**Table 2:** Description of the food components recommended by traditional Mediterranean Diet definitions and adaptations from other clinical trials and intervention studies in minority/racial populations in the United States.

Traditional Mediterranean Diet Food Components		Adaptation of the Mediterranean Diet in Minority/Racial Populations		
Trichopoulou et al. [16]	PREDIMED Study. [4]	¡Viva Bien! (Latinas with diabetes) [41]	Heart Healthy Lenoir Project (65% African Americans) [42]	*EnForma* (Hispanics-mostly Mexican) [43]
Ratio of monounsaturated to saturated lipids	Olive oil (mainly extra virgin olive oil, ≥4 tbsp/day. Using olive oil as a main culinary fat	Try to decrease the fat component of recipes. They encourage use of olive oil and vegetables oils	Include other healthy fats such as nuts, fish, full fat salad dressing and spreads. The rest of the food prepared with olive oil or vegetable oil such as avocado oil	Use healthful vegetable oils for frying, sautéing, and baking. Use full fat salad dressing and mayonnaise. Aim for 6 or more servings per week
Vegetables	Vegetables (≥2 s/day)	Emphasized consumption of fruit and vegetables	Goal to consume ≥7 s/day	Goal to consume ≥7 s/day
Fruits and nuts	Fruit (≥3 s/day) Tree nuts ≥3 s/week	Emphasized consumption of nuts	=PREDIMED study	=PREDIMED study
Legumes	Legumes (≥3 s/week)	Emphasized consumption of legumes	Eat more beans and peas. Aim for 3 or more servings per week	Eat more beans and peas. Aim for 3 or more servings per week
Dairy products			Full fat dairy products are high in saturated fat but they do not seem to increase the risk of heart disease. If you enjoy dairy products, 2–3 servings of low or full fat products is a good goal	Limit high sugar dairy products like ice cream, ice milk, and frozen yogurt to a couple times a week
Cereals		Emphasized consumption of whole grain cereals	Choose more whole grain breads. Aim for 2 or more servings of whole grain products each day	Choose more whole grain breads. Aim for 2 or more servings of whole grain products
	Sofrito	Maintaining flavor with spices and traditional ingredients	Not recommended	Not recommended
Fish	Fish and seafood (≥3 s/week)	In the cooking demonstration they tried to introduced seafood	≥1 s/week	≥1 s/week
	White meat instead of red med	Limited animal fat, and portion control.	Poultry is healthful & economical and can be eaten ≥3 times per week	Poultry is healthful & economical and can be eaten ≥3 times per week
Meat	Red meat and processed meats (limit its consumption)		Limit red meat to no more than 1 serving per day and avoid cold cuts and other processed meats	Limit red meat to no more than 1 serving per day and avoid cold cuts and other processed meats
	Limit sweets and sugar and sweetened beverages		Limit high sugar dairy products like ice cream, ice milk, and frozen yogurt to a couple times a week	Limit high sugar dairy products like ice cream, ice milk, and frozen yogurt to a couple times a week
	Butter, cream, spread fat (limit the consumption)		Use *trans*-fat free margarine instead of stick margarine	Use *trans*-fat free margarine instead of stick margarine
Alcohol (between 10 and 50 g per day and to women who consumed between 5 and 25 g per day)	Wine in moderation with meals (optional only for habitual drinkers)		Do not recommend starting wine consumption but provide information on effects of alcohol for heart health suggesting up 1 s/day for women and 2 for men	Do not recommend starting wine consumption but provide information on effects of alcohol for heart health suggesting up 1 s/day for women and 2 for men
		Include Mediterranean cooking demonstration, traditional ingredients, and common staples of Latin American. MedDiet potluck		Include revisions of culturally relevant pictures, food terms, and gastronomic preferences

Olive oil, 1 tablespoon = 13.5 g; Vegetables, 1 serving = 200 g; Tree nuts: 1 serving = 30 g; Legumes: 1 serving = 150 g; Fish and seafood: 100–150 g of fish, 4–5 pieces or 200 g seafood).
